# MRI-based random survival Forest model improves prediction of progression-free survival to induction chemotherapy plus concurrent Chemoradiotherapy in Locoregionally Advanced nasopharyngeal carcinoma

**DOI:** 10.1186/s12885-022-09832-6

**Published:** 2022-07-06

**Authors:** Wei Pei, Chen Wang, Hai Liao, Xiaobo Chen, Yunyun Wei, Xia Huang, Xueli Liang, Huayan Bao, Danke Su, Guanqiao Jin

**Affiliations:** 1 Department of Medical Imaging Center, Guangxi Medical University Cancer Hospital, No. 71, Hedi Rd, Nanning, 530021 Guangxi China; 2 Southern Medical University, Guangzhou, China; 3 Department of Radiology, Wuzhou red cross hospital, Wuzhou, Guangxi China

**Keywords:** Nasopharyngeal carcinoma, Magnetic resonance imaging, Radiomics, Machine learning, Radom survival forest

## Abstract

**Background:**

The present study aimed to explore the application value of random survival forest (RSF) model and Cox model in predicting the progression-free survival (PFS) among patients with locoregionally advanced nasopharyngeal carcinoma (LANPC) after induction chemotherapy plus concurrent chemoradiotherapy (IC + CCRT).

**Methods:**

Eligible LANPC patients underwent magnetic resonance imaging (MRI) scan before treatment were subjected to radiomics feature extraction. Radiomics and clinical features of patients in the training cohort were subjected to RSF analysis to predict PFS and were tested in the testing cohort. The performance of an RSF model with clinical and radiologic predictors was assessed with the area under the receiver operating characteristic (ROC) curve (AUC) and Delong test and compared with Cox models based on clinical and radiologic parameters. Further, the Kaplan-Meier method was used for risk stratification of patients.

**Results:**

A total of 294 LANPC patients (206 in the training cohort; 88 in the testing cohort) were enrolled and underwent magnetic resonance imaging (MRI) scans before treatment. The AUC value of the clinical Cox model, radiomics Cox model, clinical + radiomics Cox model, and clinical + radiomics RSF model in predicting 3- and 5-year PFS for LANPC patients was [0.545 vs 0.648 vs 0.648 vs 0.899 (training cohort), and 0.566 vs 0.736 vs 0.730 vs 0.861 (testing cohort); 0.556 vs 0.604 vs 0.611 vs 0.897 (training cohort), and 0.591 vs 0.661 vs 0.676 vs 0.847 (testing cohort), respectively]. Delong test showed that the RSF model and the other three Cox models were statistically significant, and the RSF model markedly improved prediction performance (*P <* 0.001). Additionally, the PFS of the high-risk group was lower than that of the low-risk group in the RSF model (*P <* 0.001), while comparable in the Cox model (*P* > 0.05).

**Conclusion:**

The RSF model may be a potential tool for prognostic prediction and risk stratification of LANPC patients.

**Supplementary Information:**

The online version contains supplementary material available at 10.1186/s12885-022-09832-6.

## Background

Nasopharyngeal carcinoma (NPC) is an epithelial malignant tumor that originates from the nasopharyngeal mucosa, characterized by distinct geographical distribution and is particularly prevalent in the south of China [1, 2]. More than 70% of NPC patients have been in locoregionally advanced stage (stage III-IVa) at diagnosis [3]. Big-data and multi-center studies have shown that compared with CCRT alone, IC + CCRT significantly improves the survival rate in LANPC patients [4, 5]. Moreover, IC + CCRT was proposed as level 2A evidence for these patients by the National Comprehensive Cancer Network (NCCN) guidelines, and it has become the first-line therapy for LANPC [6]. Nevertheless, approximately 20-30% of NPC patients report unsatisfactory efficacy after IC + CCRT [7, 8], and local recurrence and distant metastasis are still the main reasons for treatment failure in LANPC patients [9]. The application of IC + CCRT for ineffective NPC patients will significantly increase the toxicity and treatment cost [10]. Therefore, it is essential to accurately predict the treatment response, prognosis and survival of LANPC patients undergoing IC + CCRT before treatment, and to guide clinicians to develop individualized treatment regimens for patients. Further, identifying an effective prognostic prediction method is warranted for LANPC patients before IC + CCRT.

Presently, TNM staging system and MRI are routine approaches for therapeutic decision-making and prognostic prediction of LANPC [11, 12]. However, TNM staging system and traditional MRI techniques such as T1-weighted imaging (T_1_WI) and T2-weighted imaging (T_2_WI) are mainly based on the anatomical structure of tumor invasion, without considering the microscopic conditions in the tumor, which cannot accurately predict the prognosis of patients. Inflammatory biomarkers have been shown to be prognostic predictors for NPC patients. However, different study sample sizes and therapeutic approaches can lead to different cut-off values ​​of inflammatory biomarkers, limiting their predictive value for prognosis of LANPC patients [13, 14]. Radiomics is a rapidly emerging analytical approach. Radiomics analysis based on imaging data can reflect the heterogeneity within the tumor through numerous automatically extracted data characterization algorithms [15]. Tumor heterogeneity may be closely associated with cancer staging, prognostic prediction, and treatment response [16]. Recently, radiomics has been applied to predict the efficacy and prognosis of NPC, and it has shown that radiomics features are associated with PFS, recurrence, metastasis, and other clinical outcomes [17–20]. Although there are many different algorithms available for the development of radiomics risk models for NPC, it is unclear which algorithm is optimal in efficiency. The traditional Cox risk regression model is the most commonly used one for predicting the efficacy and prognosis of NPC, but it is unstable in diagnostic efficiency, and no standardized guideline is available. Thus, it remains controversial in the prognostic prediction of NPC [21–23].

The RSF model is an integrated machine learning model based on survival trees, which is suitable for the construction of prognostic models of survival data. Unlike the Cox risk regression model, this model does not need to hypothesize the distribution of parameters in advance, and the effect of variables on the risk function is linear. Hence, it is suitable for modeling high-dimensional complex data and can explore the nonlinear effects of variables on prognosis [24, 25]. In addition, the RSF model can also rank the importance of variables to screen variables with greater importance and reduce the dimensions of variables, which is conducive to the application of the model in clinical practice. Lin et al. [26] constructed an RSF model to predict the survival outcome of hepatocellular carcinoma (HCC) patients with Barcelona Clinic Liver Cancer (BCLC)-B after transcatheter arterial chemoembolization (TACE). There are also studies comparing RSF with other methods including Cox regression model, and the findings demonstrate that the performance of RSF is superior or comparable to other models [27]. In addition, the RSF model has also shown good prediction performance in the prognostic studies of tumors such as glioma and lung cancer [28, 29]. Nevertheless, few data are available regarding the accuracy of the RSF model vs the traditional Cox risk regression model in predicting the prognosis of LANPC patients after IC + CCRT.

The present study aimed to construct prediction models by RSF method and Cox regression based on clinical and radiomics parameters of LANPC patients after IC + CCRT, respectively, and compare the prediction performance of these models. It was hypothesized that the RSF model had higher performance, which would help improve the precise individualized treatment and clinical decision-making of LANPC patients.

## Materials and methods

### Study design and participants

The present study used a dataset from the medical record at our hospital from January 2015 to June 2018. Patients were eligible for inclusion if they had a histological diagnosis of LANPC, had not received any anti-tumor therapy, underwent MRI scan (including axial T_2_WI and CET_1_WI images) and IC + CCRT before treatment. The exclusion criteria were: 1) distant metastasis before the initial treatment; 2) pre-existing or concurrent malignant tumors; 3) insufficient quality of MRI due to motion artifacts or poor contrast material injection.

Eligible patients were randomly assigned to the training cohort(*n =* 206) and testing cohort(*n =* 88) at a ratio of 7:3. Tumor staging was classified according to the 8th edition of the American Joint Committee on Cancer (AJCC) TNM Staging System Manual. According to the World Health Organization (WHO) criteria, the histological tumor subtypes were classified as type I (differentiated keratinizing carcinoma), type II (differentiated non-keratinizing carcinoma), and type III (undifferentiated non-keratinizing carcinoma). The present study was approved by the Institutional Review Board, and the written informed consent was waived.

### Treatment and data collection

Details about the treatments of the patients is shown in Supplementary Materials. Patients were followed up every 1-3 months in the first 2 years, once every 6 months in the 3-5 years, and once a year thereafter. All participants were followed up for at least 2 years. The study endpoint was the PFS, which was calculated from the starting of treatment to the disease progression (or censored at the last follow-up).

### Image acquisition and segmentation

**Fig. 1:**
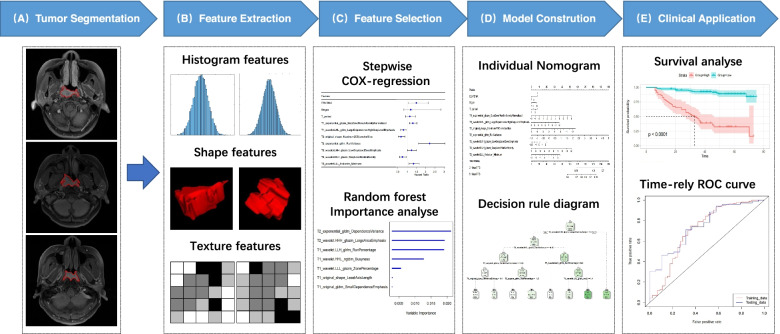
The study workflow chart. Note: The workflow for constructing radiomic features: (**A**) tumor segmentation: segmentation is made on T_2_WI and CET_1_WI images, and the experienced radiologist outlines the tumor area on each axial MRI slice; (**B**) feature extraction: the corresponding tumor features are extracted from the outlined ROI, such as histogram features, shape features, texture features, etc.; (**C**) feature selection: univariate/multivariate Cox regression method and random forest method are used to select features; (**D**) model construction: the Cox and RSF prediction models are constructed; (**E**) clinical application: The risk stratification analysis and ROC curve of the model are further applied to the clinic

The details regarding the acquisition parameters and image segmentation are presented in Supplementary Materials. The workflow chart of radiomics was shown in Fig. 1. All tumor segmentations were conducted blindly by two radiologists (observers 1 and 2 with 10 and 15 years of clinical experience in interpretation of head and neck MRI images) (Fig. 1A).

A total of 2074 radiomics features were extracted from the T_2_WI and CET_1_WI images of each patient, including histogram features, shape features, and texture features (Fig. 1B)**.** All feature parameters were standardized by Z-score based on training cohort data, and the univariate/multivariate Cox regression method and RSF method were used to reduce the dimensionality of high-dimensional data (Fig.1C) to extract the optimal features.

Construction of the Cox prediction model: Based on the multivariate stepwise Cox analysis results of clinical and radiomics features in the training cohort, the Cox prediction model of the training cohort was constructed (Fig. 1D). The model was as follows: (1) Cox model based on clinical features (clinical Cox model); (2) Cox model based on radiomics features (radiomics Cox model); and (3) Cox model based on clinical and radiomics features (clinical + radiomics Cox model); (4) RSF model based on clinical and radiomics features (clinical + radiomics RSF model). The above models were verified in the test cohort.

Construction of the RSF model: RSF was calculated by a group of binary decision trees; bootstrap and random node splitting were used to grow independent decision trees, and then all trees were set to form RSF. Details about the training steps of the RSF model is shown in Supplementary Materials. The output risk scores of the Cox and RSF models stratified patients into high- and low-risk groups based on clinical and radiomics features in the training cohort and testing cohort; and the survival outcome between the high-risk group and the low-risk group was compared.

### Statistical analysis

Statistical analyses were performed with the use of R software (4.1.1). Normally distributed measurement data were presented as mean ± standard deviation (SD) and compared by the t test; measurement data of skewed distribution were presented as M (range) and compared by the Mann-Whitney U test. Count data were presented as absolute number or percentage and compared using the χ^2^ test. Univariable and multivariable survival analyses were conducted using the Cox proportional hazards model. The Kaplan-Meier method was used to plot the survival curve and the survival rate was calculated; the X-tile software was used to select the optimal cut-off value for continuous variables, and the log-rank test was conducted to compare whether the difference in survival time between the two groups was statistically significant. All tests were two-tailed with significance tests, and *P <* 0.05 was considered statistically significant. A time-dependent ROC curve was plotted, and the AUC was calculated to evaluate the prediction performance of different models. The Delong test was used to compare the performance among models. To ensure the stability of the testing effect, the prediction model of the training cohort was confirmed in the testing cohort.

## Results

### Clinical characteristics of the patients

A total of 294 patients (213 males and 81 females; the mean age was 43.6 years (SD: 10.9 years, range: 19-71 years) were enrolled in the present study. The last follow-up ended on May 21, 2021, and the median follow-up time was 43.9 months (range:8.0-75.0 months). The clinical characteristics of all LANPC patients in the training cohort and testing cohort were summarized in Table 1. Univariate and multivariate Cox regression analyses were used to explore the clinical characteristics, and the results showed that Epstein-Barr virus (EBV) DNA, Overall Stage, and T stage were independent risk factors that affected the survival and prognosis of NPC patients (all *P <* 0.05) (Table 2).

**Table 1:** Clinical characteristics of the patients

Variable		Training cohort			Testing cohort		
		Label = 0	Label = 1	***P*** value	Label = 0	Label = 1	***P*** value
		***N =*** 153	***N =*** 53		***N =*** 65	***N =*** 23	
Age, years		45.00 [34.00, 51.00]	48.00 [35.00, 52.00]	0.412	43.00 [37.00, 50.00]	45.00 [39.00, 52.50]	0.283
Height		1.65 [1.60, 1.70]	1.65 [1.60, 1.70]	0.674	1.65 [1.60, 1.70]	1.62 [1.54, 1.65]	0.023
Weight, kg		60.00 [53.00, 66.50]	59.00 [52.50, 65.00]	0.523	65.00 [57.00, 69.00]	54.00 [49.25, 61.75]	0.006
BMI		22.46 [20.08, 24.22]	21.97 [19.33, 24.22]	0.329	23.44 [20.90, 25.39]	21.09 [19.83, 23.66]	0.052
Follow-up Time, month		49.00 [37.00, 59.00]	20.00 [11.00, 33.00]	< 0.001	46.00 [37.00, 58.00]	19.00 [13.00, 24.50]	< 0.001
Sex (%)	Female	44 (28.8)	11 (20.8)	0.256	20 (30.8)	6 (26.1)	0.672
	Male	109 (71.2)	42 (79.2)		45 (69.2)	17 (73.9)	
Family (%)	No	123 (80.4)	44 (83.0)	0.674	52 (80.0)	16 (69.6)	0.305
	Yes	30 (19.6)	9 (17.0)		13 (20.0)	7 (30.4)	
Smoke (%)	No	90 (58.8)	32 (60.4)	0.843	40 (61.5)	11 (47.8)	0.252
	Yes	63 (41.2)	21 (39.6)		25 (38.5)	12 (52.2)	
EBV-DNA (%)	0	88 (57.5)	26 (49.1)	0.286	33 (50.8)	11 (47.8)	0.808
	1	65 (42.5)	27 (50.9)		32 (49.2)	12 (52.2)	
T stage (%)	T1	8 (5.2)	3 (5.7)	0.355	1 (1.5)	0 (0.0)	0.473
	T2	38 (24.8)	8 (15.1)		18 (27.7)	5 (21.7)	
	T3	40 (26.1)	12 (22.6)		22 (33.8)	5 (21.7)	
	T4	67 (43.8)	30 (56.6)		24 (36.9)	13 (56.5)	
N stage (%)	N0	3 (2.0)	0 (0.0)	0.363	1 (1.5)	0 (0.0)	0.307
	N1	36 (23.5)	18 (34.0)		14 (21.5)	7 (30.4)	
	N2	67 (43.8)	23 (43.4)		23 (35.4)	11 (47.8)	
	N3	47 (30.7)	12 (22.6)		27 (41.5)	5 (21.7)	
Overall stage (%)	3	51 (33.3)	16 (30.2)	0.674	21 (32.3)	6 (26.1)	0.578
	4	102 (66.7)	37 (69.8)		44 (67.7)	17 (73.9)	
WHO Grade (%)	I	2 (1.4)	1 (1.9)	0.422	2 (3.0)	0 (0.0)	0.558
	II	19 (12.4)	10 (18.9)		6 (9.2)	0 (0.0)	
	III	132 (86.3)	42 (79.2)		57 (87.7)	23 (100.0)	
WBC		7.22 [6.21, 8.76]	6.88 [5.53, 7.99]	0.079	7.17 [6.21, 8.49]	6.91 [5.70, 8.18]	0.35
Hb		137.00 [126.00, 150.00]	138.00 [125.00, 148.00]	0.646	140.00 [129.00, 149.00]	132.00 [119.50, 143.00]	0.072
PLT		272.00 [242.00, 332.00]	289.00 [231.00, 337.00]	0.934	286.00 [234.00, 335.00]	296.00 [238.00, 325.00]	0.966
NEUT		4.51 [3.55, 5.70]	4.39 [3.53, 5.70]	0.621	4.45 [3.60, 5.55]	3.91 [3.20, 4.94]	0.086
LYMP		1.87 [1.62, 2.24]	1.76 [1.42, 2.10]	0.076	1.88 [1.53, 2.32]	2.00 [1.63, 2.53]	0.403
ALB		0.06 (1.16)	−0.10 (0.90)	0.377	−0.11 [−0.48, 0.43]	−0.18 [−0.46, 0.29]	0.966

Note: Normally distributed measurement data are presented as mean ± SD and compared by the t test; measurement data of skewed distribution are presented as M (range) and compared by the Mann-Whitney U test. Count data are presented as absolute number or percentage and compared using the χ^2^ test. If the chi-square test conditions are not met, the exact probability test should be used

Abbreviations and definitions: Label (0, No disease progression or death from any cause; 1, First occurrence of disease progression or death from any cause); *BMI* Body mass index, *EBV-DNA* Epstein-Barr virus DNA (0, < 1000 copies/ml; 1, ≥1000 copies/ml), *T* Tumor, *N* Lymph node, *WHO Grade* WHO pathological subtypes of nasopharyngeal carcinoma [type I (differentiated keratinizing carcinoma), type II (differentiated non-keratinizing carcinoma), and type III (undifferentiated non-keratinizing carcinoma)], *WBC* White blood cell, *Hb* Hemoglobin, *PLT* Platelet, *NEUT* Neutrophil count, *LYMP* Lymphocyte count, *ALB* Albumin

**Table 2:** Univariate and multivariate Cox regression analysis

		Univariate cox regression		Multivariate cox regression	
	Factors	HR (95% CI)	*P* value	HR (95% CI)	*P* value
1	Age	0.993 (0.981-1.005)	0.273	–	–
2	ALB	0.928 (0.812-1.06)	0.268	–	–
3	BMI	1.015 (0.98-1.05)	0.402	–	–
4	EBV-DNA	1.605 (1.225-2.101)	0.001	1.739 (1.248-2.421)	0.001
6	Family	0.922 (0.66-1.289)	0.634	–	–
7	WHO Grade	1.317 (0.968-1.791)	0.079	–	–
8	Hb	0.999 (0.991-1.008)	0.89	–	–
9	Height	1.121 (0.974-1.29)	0.11	–	–
10	LYMP	1.052 (0.839-1.32)	0.66	–	–
11	NEUT	0.995 (0.93-1.065)	0.89	–	–
12	N stage	1.025 (0.864-1.216)	0.779	–	–
13	PLT	1 (0.998-1.002)	0.945	–	–
14	Sex	0.747 (0.557-1.003)	0.053	–	–
15	Smoke	1.124 (1.475-0.856)	0.399	–	–
16	Overall stage	1.318 (1.013-1.715)	0.04	1.272 (1.042-1.553)	0.019
17	T stage	1.179 (1.022-1.361)	0.024	1.676 (1.079-2.604)	0.022
18	WBC	1.007 (0.949-1.069)	0.808	–	–
19	Weight	1.005 (0.994-1.016)	0.382	–	–

*Abbreviations and definitions*: *ALB* Albumin, *BMI* Body mass index, *EBV-DNA* Epstein-Barr virus DNA, *WHO Grade* WHO pathological subtype of nasopharyngeal carcinoma, *Hb* Hemoglobin, *LYMP* Lymphocyte count, *NEUT* Neutrophil count, *N* Lymph node, *PLT* Platelet, *T* Tumor, *WBC* White blood cell

### Construction of radiomics labelling

The ICC values between the features of the two observers and the ICC value of the features extracted by the ROI plotted by the observer A were calculated for comparison. Among them, the repeatability between the two features based on the observer A was excellent (ICC = 0.782-0.957), and the consistency of the features between the two observers was good (ICC = 0.732-0.948). In the 2074 radiomics features extracted from T_2_WI and CET_1_WI images, radiomics labeling was constructed by univariate and multivariate stepwise Cox analysis.

### Construction and verification of the cox nomogram model

**Fig. 2:**
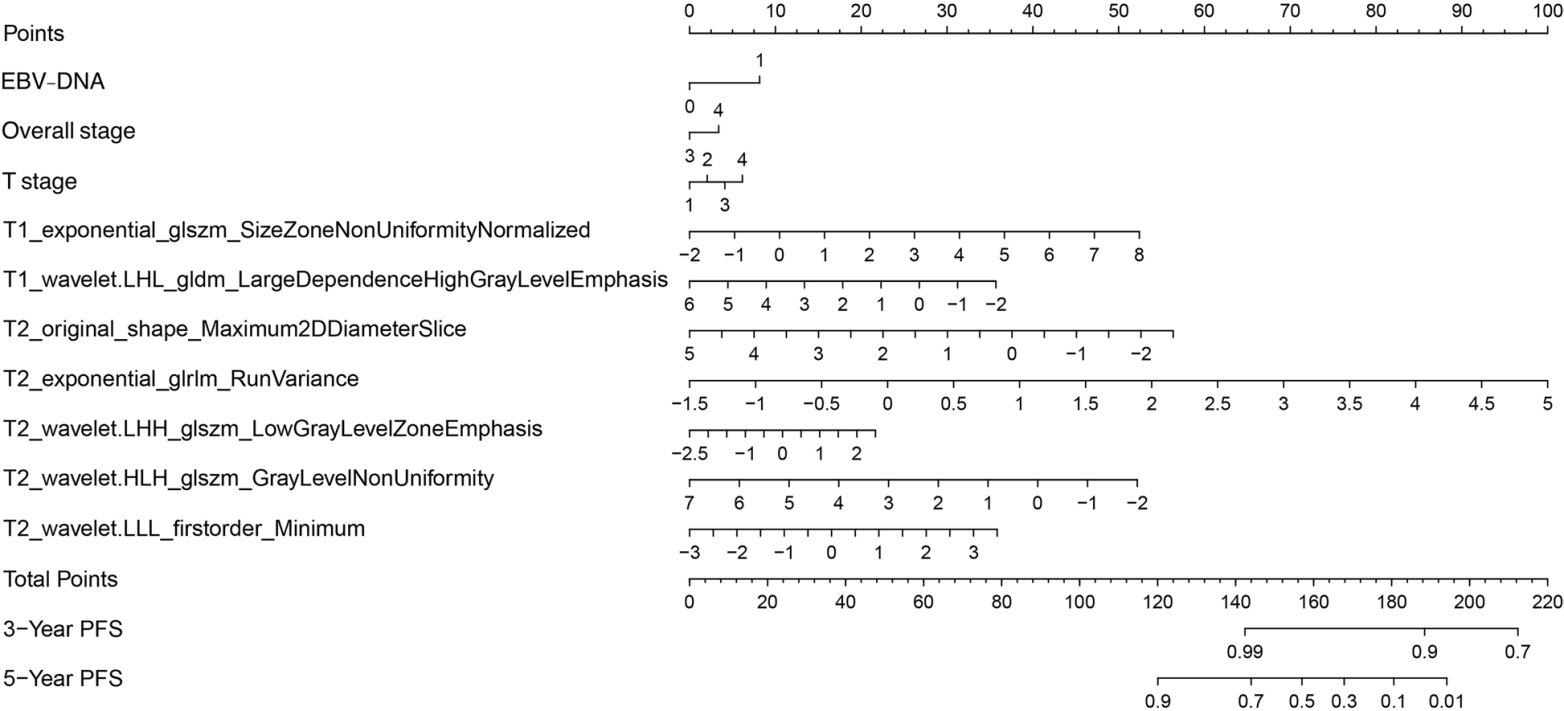
Visual nomogram of the clinical + radiomic Cox model in predicting 3- and 5-year PFS. Note: EBV-DNA, Epstein-Barr virus DNA (0, < 1000 copies/ml; 1, ≥1000 copies/ml). Nomogram is used: First, all predictor nodes can be found on the “node” line (EBV-DNA < 1000 copies/ml is rated 0 point, and EBV-DNA ≥ 1000 copies/ml 7.5 points; overall stage 3 is rated 0 point, and the overall stage 4 3.0 points; stage T1 is rated 0 points, stage T2 2.0 points, stage T3 4.0 points, and T4 6.0 points, and so on) . Then ten predicted nodes are added to the “total score” row. Finally, a vertical line was plotted down from the “total score” to the “3- or 5-year survival rate” axis

**Fig. 3:**
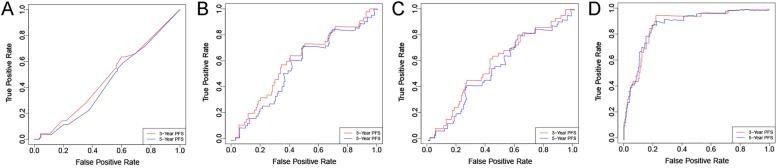
ROC curve of each model in the training cohort. Note: **A** ROC curve of clinical Cox model; **B** ROC curve of radiomics Cox model; **C** ROC curve of clinical + radiomics Cox model; **D** ROC curve of clinical + radiomics RSF model

**Fig. 4:**
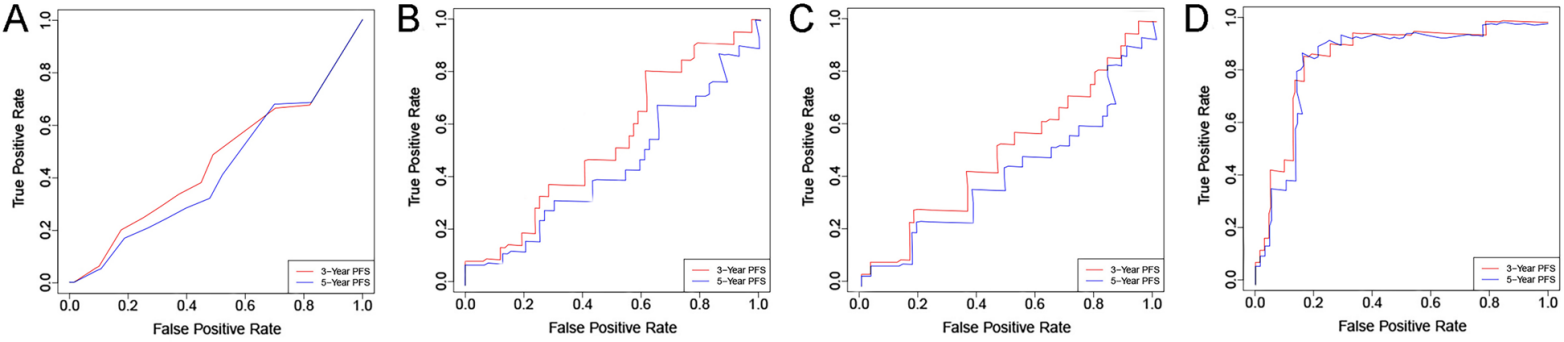
ROC curve of each model in the testing cohort. Note: **A** ROC curve of clinical Cox model; **B** radiomics Cox model; **C** ROC curve of clinical + radiomics Cox model; D ROC curve of clinical + radiomics RSF model

A nomogram was constructed based on significant variables in univariate and multivariate Cox analyses (these variables are presented in Supplementary Materials). In the current nomogram (Fig. 2), a node was assigned to each variable based on HR. By adding up the total scores of each variable and positioning it on the total score scale, the probability of 3- and 5-year PFS were obtained. In the training cohort, the AUC of the clinical Cox model, the radiomics Cox model, and the clinical + radiomics Cox model in predicting the 3-year PFS after NPC treatment was 0.545, 0.648, and 0.648, respectively; the AUC of 5-year PFS was 0.556, 0.604, and 0.611, respectively. In the testing cohort, the AUC of the three models in predicting the 3-year PFS after NPC treatment was 0.566, 0.736, and 0.730, respectively; the AUC of 5-year PFS was 0.591, 0.661, and 0.676, respectively. The ROC curve was shown in Figs. 3 and 4. Overall, in the comparison among the three Cox models, the prediction performance was comparable (Table 3).

**Table 3:** AUC results of the models

AUC		clinical Cox model	radiomics Cox model	Clinical + radiomics Cox model	Clinical + radiomics RSF model
Training	3-Year PFS	0.545	0.648	0.648	0.899
	5-Year PFS	0.556	0.604	0.611	0.897
Testing	3-Year PFS	0.566	0.736	0.73	0.861
	5-Year PFS	0.591	0.661	0.676	0.847

### Construction and verification of the RSF model

**Fig. 5:**
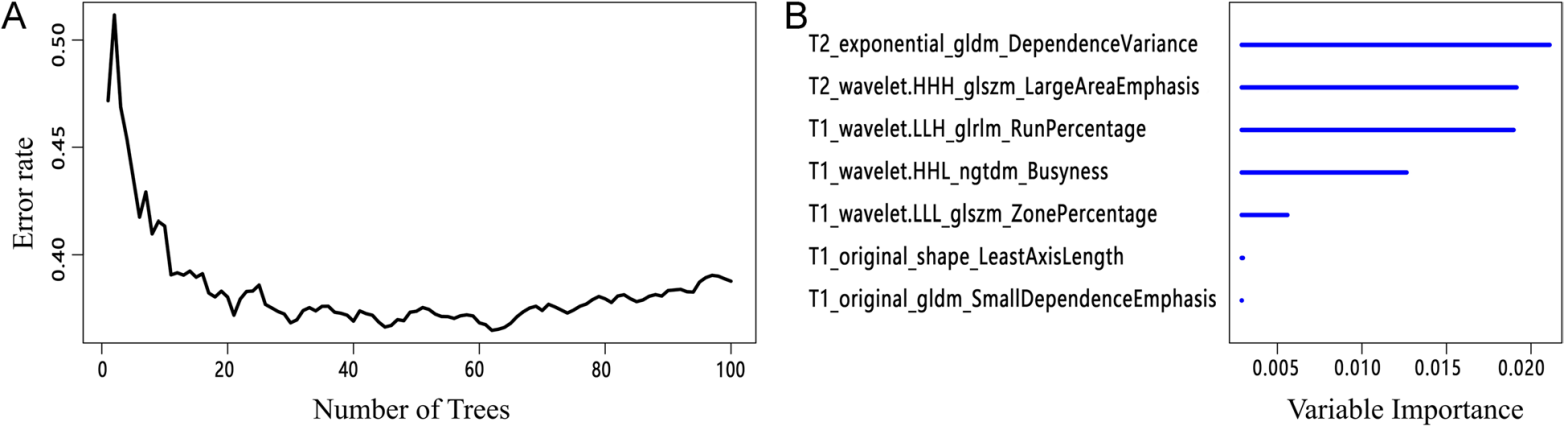
Curve chart of the error rate of the RSF model and importance bar chart of the most important features. Note: **A** Curve chart of the error rate of the RSF model. The abscissa is the number of survival trees, and the ordinate is the error rate of the model in the training set. It can be observed that when there are more than 20 trees in the forest, the error rate tends to be stable and maintains around 0.1-0.3. **B** Importance bar chart of the most important features. The importance order of the most important radiomics features for the RSF model in predicting the PFS. The RSF model is constructed according to the optimal parameter ntree to obtain the importance of each predictive variable, and sorting is conducted based on the importance score in the order of the largest to the smallest

**Fig. 6:**
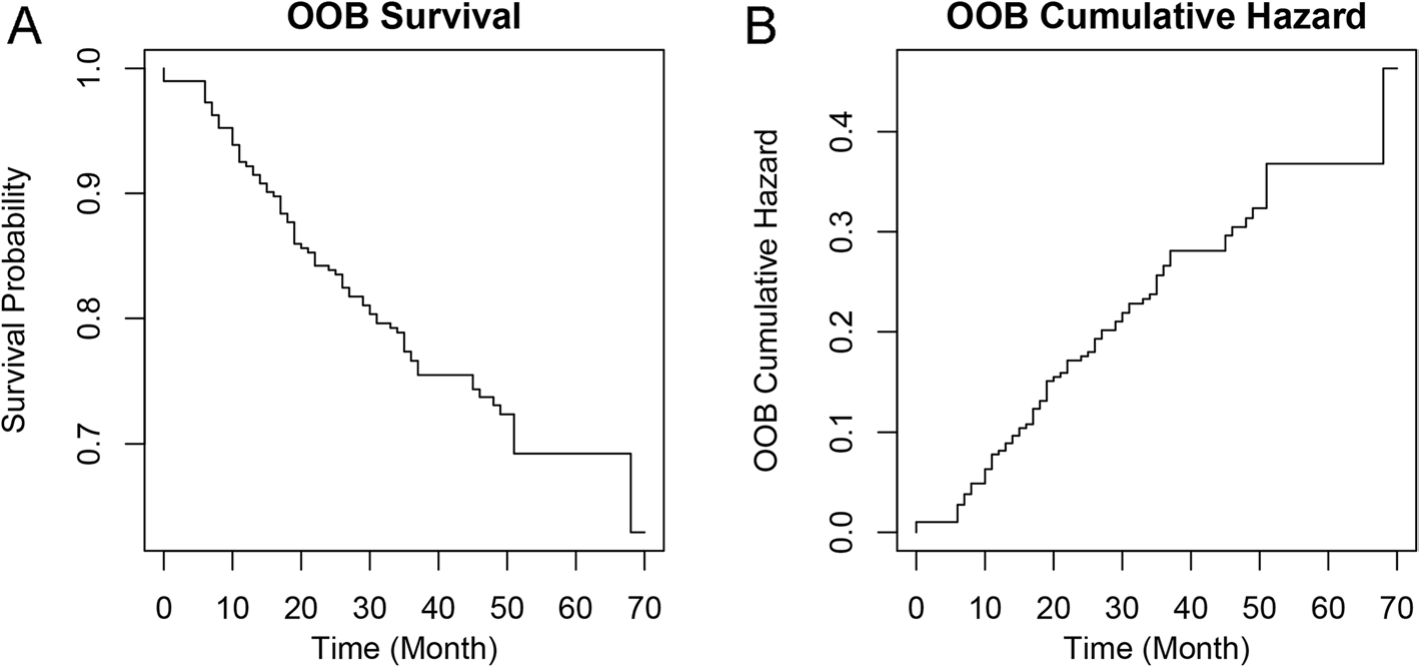
Survival rate curve and cumulative hazard curve: for predicting PFS in LANPC patients. Note: **A** Survival rate curve; **B** Cumulative hazard curve

**Fig. 7:**
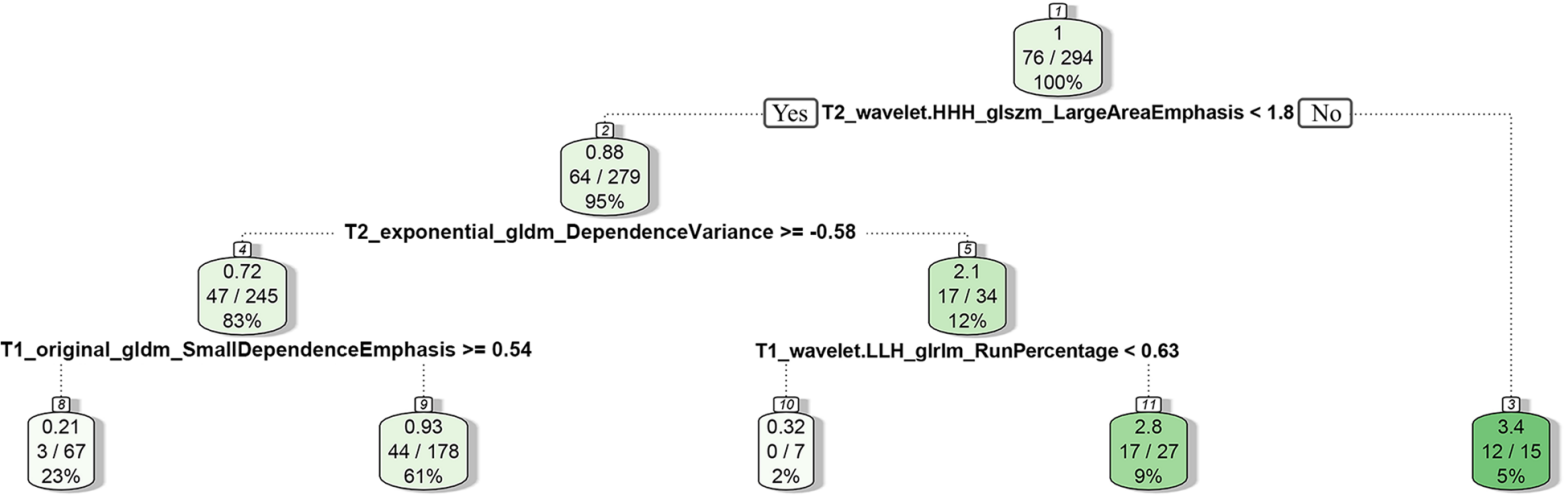
Decision rule of the RSF (Taking the tree depth of 4 (depth = 4) as an example). Note: The positive samples in the initial training set sample account for 76/294, which are continuously split according to the split rule of the index below the jade pendant icon. If the condition is met (yes), it will be extended to the left, and if the condition is not met (no), it will be extended to the right. After each split, 2 sub-data sets can be obtained. When the expected depth (depth = 4) is reached, the model stops splitting

The error rate corresponding to the number of survival trees within 100 was obtained, as shown in Fig. 5. The results showed that when constructing 100 survival trees, the error rate was low and maintained a relatively stable level. The RSF model was constructed according to the optimal parameter ntree = 100, and as it shows in Fig. 5 and in Supplementary Materials, 7 features associated with the PFS were selected according to the importance score of each radiomics feature. The survival rate and cumulative hazard curves plotted over time were shown in Fig. 6. The results showed that as the survival time increased, the prediction performance of the RSF model in the survival rate gradually decreased, and the cumulative hazard increased. The decision rule diagram based on the RSF model was shown in Fig. 7.

**Fig. 8:**
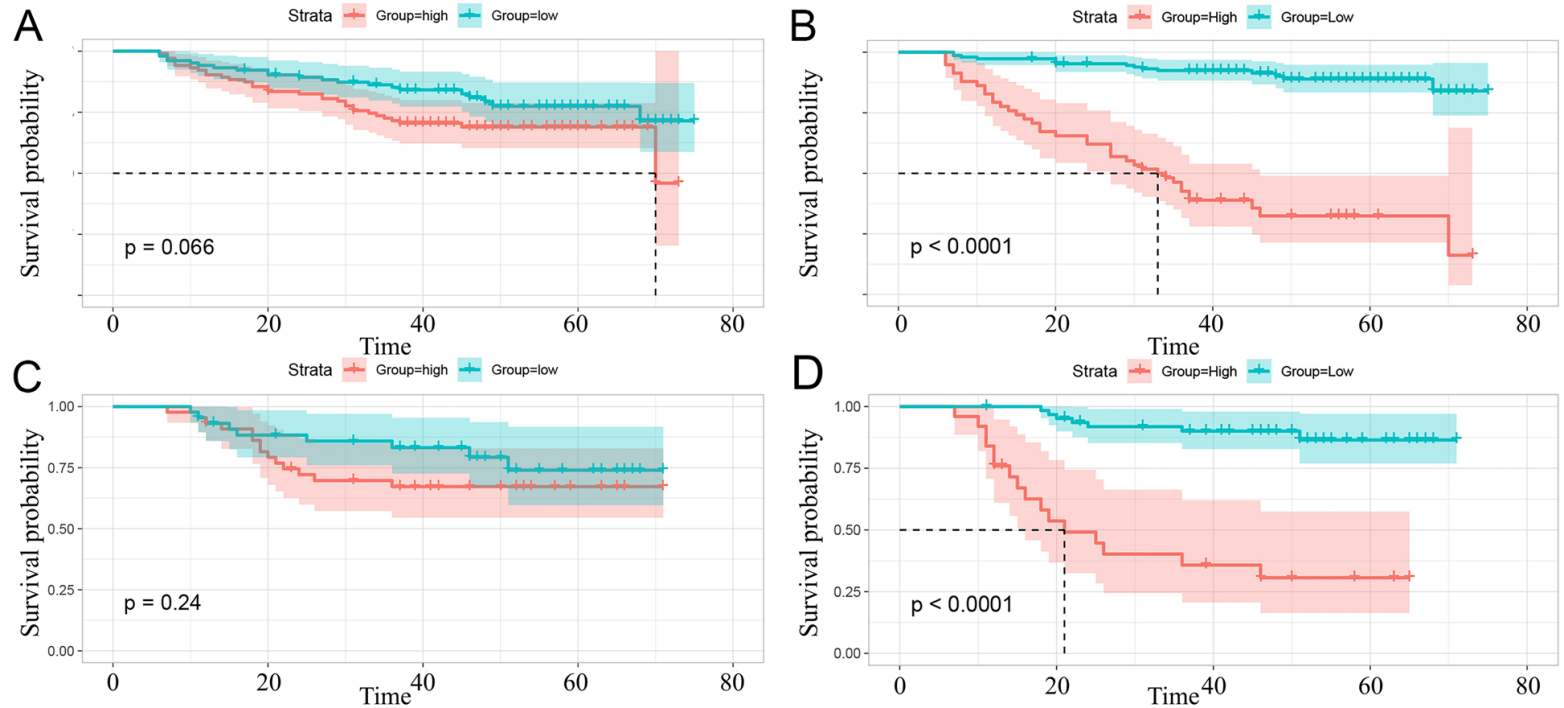
Kaplan-Meier curves of different stratification methods. Note: The Kaplan–Meier survival analysis is conducted to estimate the high- and low-risk PFS in the training and testing cohorts. **A** risk stratification of the clinical + radiomics Cox model in the training cohort; **B** risk stratification of the RSF model in the training cohort; **C** risk stratification of the clinical + radiomics Cox model in the testing cohort; D risk stratification of the RSF model in the testing cohort

In the training cohort, the AUC of the RSF model in predicting the 3- and 5-year PFS after NPC treatment was 0.899 and 0.897, respectively; in the testing cohort, it was 0.861 and 0.847, respectively. Compared with the three Cox models, the RSF model showed the highest prediction performance, and the differences among the models were statistically significant (all *P* < 0.001,Table 4). Patients in the low-risk group achieved better PFS (all *P <* 0.001,Fig. 8), demonstrating the good clinical application value of this model.

**Table 4:** Performance comparison among the models-Delong test

DeLong. Test	clinical Cox model	radiomics Cox model	Clinical+radiomics Cox model	Clinical+radiomics RSF model
clinical Cox model	/	0.797 ^b^	0.971 ^b^	< 0.001 ^b^
radiomics Cox model	0.436 ^a^	/	0.539 ^b^	< 0.001 ^b^
clinical+radiomics Cox model	0.280 ^a^	0.551 ^a^	/	< 0.001 ^b^
clinical+radiomics RSF model	< 0.001^a^	< 0.001 ^a^	< 0.001 ^a^	/

^a^ Training set; ^b^ Testing set

### Stratification analysis of the clinic + radiomics cox nomogram model and RSF model

According to the ROC curves of the Cox and RSF models in the training set, the prognostic risk score maximizing the Youden index was used as the threshold (cutoff value), which was used to assign patients to the non-high-risk group (the prognostic risk score was less than the threshold) and high-risk group (the prognostic risk score was greater than or equal to the threshold). Figure 8 showed the Kaplan-Meier survival curves of the two models, which were used to stratify patients into high- and low-risk groups based on risk scores for treatment recommendations. Kaplan-Meier survival analysis showed that Cox combination model could not distinguish PFS in high- and low-risk patients (*P* > 0.05; Fig. 8A and C), whereas the RSF model could distinguish PFS in high- and low-risk patients (*P* < 0.001; Fig. 8B and D).

## Discussion

In the present study, two different models were constructed to predict the PFS of LANPC patients after IC + CCRT. The current findings suggested that compared with the conventional Cox model, the RSF model significantly improved the predictive value and successfully distinguished high-risk and low-risk patients, indicating that it can be used as a noninvasive and useful tool for predicting the prognosis of LANPC patients.

Previous studies have demonstrated that EBV-DNA and TNM staging indicators can help predict the prognosis of NPC [30, 31]. The present multivariate analysis showed that EBV-DNA, T staging and overall stages before treatment were valuable in predicting PFS in LANPC patients, which was consistent with previous findings [3, 30, 31], so they were included in the prediction model. However, the prediction performance of the Cox model based only on clinical features was relatively low. In the training cohort, the AUC of the clinical model in predicting the 3- and 5-year PFS was 0.545 and 0.556, respectively; in the testing cohort, it was 0.566 and 0.591, respectively. The reasons may be as follows: First, patients are only in stage III-IVa, and the clinical stages are narrow and similar. Therefore, it will be more difficult to predict the PFS by clinical stages; second, the T and N stages of the present study are unbalanced, and there are only 5.2% T1 and 2.0% N0 patients in the training set. Even if the clinical staging is effective, it will produce large errors; third, the T staging and overall stages are based on the gross anatomical information of the tumor, and unable to reflect the heterogeneity within the tumor. Thus, despite the addition of EBV-DNA, the prediction performance of the model is still low.

Recently, radiomics has become a popular approach for tumor prognostic prediction. By the analysis of the whole tumor lesions, radiomics has successfully transformed medical imaging into excavated, quantitative, and high-dimensional imaging features and reflects the heterogeneity of tumors to help patients assess risks and guide clinical decision-making [32, 33]; it is a non-invasive, effective, and reliable approach. Therefore, radiomics labelling can be a useful supplement to clinical features in terms of prognostic value, which can explain the prognostic prediction performance of the radiomics model in the present study is better than that of the clinical model. The potential clinical value of predictive models based on radiomics in predicting PFS in NPC patients has been previously emphasized [21, 34]. However, previous reports mostly used the Cox model to predict the prognosis of NPC. Different studies included different stages and treatment methods for NPC patients, resulting in different clinical and radiomics features, thereby increasing the study heterogeneity and affecting the prediction performance [21–23]. A study [35] constructed a Cox proportional hazard regression model to predict the PFS of NPC patients. However, as compared with the clinical Cox model alone or staging Cox model alone, the Cox model based on radiomics did not improve survival prediction (in the training cohort, the time-dependent AUC of the radiomics Cox model, clinical Cox model, and staging Cox model was 0.71 vs 0.72 vs 0.70, respectively). Similarly, in the present study, the Cox model 3 with the addition of radiomics did not significantly improve the prognostic prediction of LANPC patients. In addition, when comparing survival differences among groups, the Cox model requires data to meet the precondition of proportional hazard hypothesis [36]. When the data does not meet the prerequisite requirements, it should make the data meet the hypothesis through stratification or data conversion for analysis. At present, many researchers ignore the testing of the proportional hazard hypothesis when using the Cox regression model, affecting the authenticity and reliability of the findings.

In the present study, based on the RSF model, the survival prediction study of LANPC patients after IC + CCRT was conducted. The findings showed that, as compared with the traditional Cox model, the RSF model significantly improved the prediction performance for PFS of LANPC, and the model had better stability. It is reported in the literature that the RSF model has the advantages of general Random forest (RF) and can prevent the overfitting of its algorithm through two random sampling processes [24]. At the same time, the advantage of the RSF model is that it is not limited by conditions such as proportional hazard and log-linear hypotheses [37]. Compared with traditional survival analysis methods such as the Cox model, the prediction accuracy of the RSF model is at least equal to or better than that of traditional survival analysis methods. Several studies have emphasized the important role of RF classifiers in the selection of radiomics features and model construction of NPC patients [38–40], which improves the accuracy of survival prediction. Previous studies [28] reported that compared with models that included clinical or genetic features alone, the RSF model with the addition of radiomics to clinical and genetic features significantly improved the survival prediction of gliomas. Another study obtained radiomics features from CT images of 573 patients with non-small cell lung cancer and fitted the RSF model, revealing that the RSF model had the potential to predict distant metastasis in patients with non-small cell lung cancer [41]. It suggests that the RSF model has a good potential for predicting the prognosis of cancer patients. Therefore, the RSF model of the present study achieved better effects in both the PFS prediction and risk stratification of LANPC patients. To our knowledge, there are few feasibility studies to explore the prognosis of LANPC patients after IC + CCRT by comparing two radiomics-based models, so the present study may be an important reference because it compared the prediction performance of different models in the training cohort and testing cohort. Such comparative studies may improve the reliability of predictive analysis models based on radiomics and help broaden the scope of radiomics in cancer treatment.

In addition, the RSF model based on clinical and radiomics features showed better prognostic prediction performance than the Cox model. The Kaplan-Meier survival curve was used to separate the patients. The PFS of the high-risk group was lower than that of the low-risk group, which was similar to previous findings [23, 32, 34, 40]; it demonstrates a significant difference between the two models, which may help to accurately stratify individual treatment strategies in clinical practice, thereby improving the clinical outcome of LANPC patients.

The present study has several limitations. First, the single-center study may limit the applicability of the present findings for patients in other regions and centers, so it needs to be further verified by multiple centers. Second, the present study only extracts the radiomics features of the primary tumor and does not explore the lymph nodes. Further, N stage was not significantly associated with prognosis. This may be related to the small number of cases in this study. In addition, due to the retrospective nature, there may be selection bias. Thus, the well-designed prospective studies are warranted.

In conclusion, the present study demonstrates that as compared with the Cox model, the RSF model including clinical and radiomics features shows better performance in predicting the PFS of LANPC patients after IC + CCRT. The RSF model can divide patients into low-risk and high-risk groups, and it may offer additional information for individual treatment strategies for LANPC patients. The construction and comparison of different radiomics prediction models will facilitate the application of radiomics in tumor precision medicine and clinical practice.

## Supplementary Information

**Additional file 1.**

## Data Availability

The datasets used during the current study are available from the corresponding author on reasonable request.

## Abbreviations

RSF: Random survival forest
NPC: Nasopharyngeal carcinoma
LANPC: Locoregionally advanced nasopharyngeal carcinoma
IC + CCRT: Induction chemotherapy plus concurrent chemoradiotherapy
MRI: Magnetic resonance imaging
ROC: Receiver operating characteristic
AUC: Areas under ROC curve
NCCN: National Comprehensive Cancer Network
HCC: Hepatocellular carcinoma
TACE: Transcatheter arterial chemoembolization
BCLC: Barcelona Clinic Liver Cancer
AJCC: American Joint Committee on Cancer
WHO: World Health Organization
IMRT: Intensity-modulated radiotherapy
TR: Time of Repetition
TE: Time of Echo
PACS: Picture archiving and communication system
ICC: Interclass correlation coefficient
OOB data: Out-of-bag data
CHF: Cumulative hazard function
VIMP: Variable importance
HR: Hazard ratio
RF: Random survival
