# Supplementary material for: MRI-based random survival Forest model improves prediction of progression-free survival to induction chemotherapy plus concurrent Chemoradiotherapy in Locoregionally Advanced nasopharyngeal carcinoma
Source: BMC Cancer. 2022 Jul 6;22:739. doi: 10.1186/s12885-022-09832-6 (PMC9261049; doi:10.1186/s12885-022-09832-6)
Supplement: Supplementary file 1 — Additional file 1. [file 12885_2022_9832_MOESM1_ESM.doc]

**Treatment**

All patients underwent IC+CCRT. The IC regimen used GP (gemcitabine 1000 mg/m^2^, d1, 8; cisplatin 80 mg/m^2^, d1-d2), TPF (docetaxel 60 mg/m^2^, d1; cisplatin 60 mg/m^2^, d1; 5-fluorouracil 600 mg/m^2^, continuous infusion for 120 h), once every 3 weeks, for 3 cycles in total. The CCRT regimen included cisplatin (40 mg/m², 5 days/week, once every 3 weeks, for 3 cycles in total), which was started from the first day of radiotherapy. Intensity-modulated radiotherapy (IMRT) was used for radiotherapy. The dose range of the primary focus was 66-72 Gy, and that of the prophylactic irradiation area was 54-56 Gy; the number of segmentation was 30-33 times.

**Image acquisition and segmentation**

All patients in the present study used a 1.5T MRI system (Magnetom Avanto, Siemens Healthcare, Erlangen, Germany) to obtain MR images. The acquisition sequences included: （1）Scanning: cross-sectional T1WI and T2WI, coronal T2WI, and sagittal T1WI. （2）Contrast-enhanced scanning: CET1WI in transverse, coronal, and sagittal planes, of which fat suppression imaging was performed in one section. The parameters were as follows: T1WI[repetition time (TR) =450 ms, echo time (TE) =15 ms], T2WI (TR =6000 ms, TE= 95 ms), FOV = 230 mm×230 mm, matrix size = 512×168, flip angle =90°, slice thickness = 5 mm, spacing between slices =0.5 mm. The contrast agent, Gd-DTPA (Magnevist meglumine, Bayer Health Care Pharmaceuticals, Germany), was injected at a dose of 0.1 mmol/kg body weight (flow rate of 2.0 mL/s).

All MR images extracted from the picture archiving and communication system (PACS) were loaded into the ITK-SNAP software version 3.6.0 (open-source software; https://itk.org/). All tumor segmentations were conducted blindly by two radiologists with experience in reading head and neck MRI images, and the tumor was manually segmented on the axial T_2_WI and CE T_1_WI image slices (Fig. 1a). Regarding repeatability evaluation, the interclass correlation coefficient (ICC) was used to evaluate the consistency of radiomics features among observers. One radiologist (Observer A, with 10 years of experience in interpretation of head and neck MRI images) plotted lesions on 50 randomly selected patients within 1 week, and then 100 radiomics features were selected from each patient (50 features were randomly selected from radiomics features based on T_2_WI, and 50 from radiomics features based on CET1-w). One month later, the radiologist (Observer A) and another radiologist (Observer B, 15 years of experience in interpretation of head and neck MRI images) performed ROI plotting and feature extraction. The independent Kruskal–Wallis H test was used to evaluate the difference between the features extracted by the Observer A (first time) and those extracted by the Observer B, as well as the two features extracted by the Observer A. When ICC was greater than 0.75, it was considered to have good repeatability and consistency.

**Construction of RSF models**

The RSF model training steps were as follows:

i. The model used Bootstrap to randomly select N-tree self-service sample sets as training samples from the original data with replacement and excluded 37% of the data in each sample as out-of-bag data (OOB data) .

ii. For each sample set, a binary recursive survival tree was constructed. It was hypothesized that the variable of the original data was *P*, mtry (mtry<*P*) candidate variables were randomly selected at each node of each tree during the construction. The candidate variable with the largest survival difference among child nodes was used as the node to split, and the difference in survival outcome was measured by log-rank test statistics.

iii. This step was followed until ntree survival trees were generated to form a survival forest. The parameters were set as follows: ntree=100 (setting 100 trees to form a forest, namely 100 classifiers, and the final prediction result was obtained by voting based on the majority rule) split rule = “log-rank” (The tree branch splitting rule was logarithmic order of importance, log-rank) to obtain 100 trees. The tree grew under the condition that the terminal node was no less than node size events, otherwise the tree stopped growing.

iv. The OOB data obtained in the random forest can calculate the prediction error rate of the integrated cumulative hazard function (CHF). A CHF was calculated for each tree, and finally the average value was obtained as the integrated CHF. The variables of radiomics features were selected according to the variable importance (VIMP). The importance score was an evaluation index used to measure the prediction performance of the predictor variable to the outcome variable. The larger the value of VIMP, the better the prediction performance. The RSF model was constructed according to the optimal parameter ntree to obtain the importance of each predictor variable, and sorting was conducted based on the importance score in the order of the largest to the smallest.

**Significant variables in univariate and multivariate Cox analyses**

Significant clinical measures (3) and imaging characteristics (7) were obtained using univariate and multivariate stepwise Cox analysis, as follows:

1. EBV-DNA.
2. Overall stages.
3. T stage.
4. T1_exponential_glszm_SizeZoneNonUniformityNormalized.
5. T1_wavelet.LHL_gldm_LargeDependenceHighGrayLevelEmphasis.
6. T2_original_shape_Maximum2DDiameterSlice.
7. T2_exponential_glrlm_RunVariance.
8. T2_wavelet.LHH_glszm_LowGrayLevelZoneEmphasis.
9. T2_wavelet .HLH_glszm_GrayLevelNonUniformity.
10. T2_wavelet.LLL_firstorder_Minimum

The above-mentioned significant clinical and radiomics features were incorporated into the model to construct a nomogram.

**Significant variables in RSF Model**

The RSF model was constructed according to the optimal parameter ntree=100, and 7 features associated with the PFS were selected according to the importance score of each radiomics feature (Fig. 5); the results showed that the radiomics feature ranking first in importance was T2_exponential_gldm_DependenceVariance, followed by T2_wavelet.HHH_glszm_LargeAreaEmphas, T1_wavelet.LLH_glrlm_RunPercentage, T1_wavelet.HHL_ngtdm_Busyness, T1_wavelet.LLL_glszm_Zone Percentage. The least important radiomics feature were T1_original_shape_LeastAxisLength and T1_original_gldm_SmallDependenceEmphasis.
